# Identification of an intramuscular gastric subepithelial stromal tumor during endoscopic resection by using endoscopic ultrasound within the submucosal tunnel

**DOI:** 10.1055/a-2764-4702

**Published:** 2026-01-20

**Authors:** Leandro Corradino, Dario Biasutto, Benedetto Neri, Serena Stigliano, Cristina Lucidi, Valeria DʼOvidio, Francesco Maria Di Matteo

**Affiliations:** 1 220431 Therapeutic GI Endoscopy Unit, Fondazione Policlinico Universitario Campus Bio-Medico, Rome, Italy; 2 9309 Department of Medicine and Surgery, University of Perugia, Perugia, Italy; 3 Unit of Gastroenterology and Digestive Endoscopy, S. Eugenio Hospital, Rome, Italy

Gastrointestinal stromal tumors (GISTs) are the most common mesenchymal tumors of the gastrointestinal tract 1 . The management includes endoscopic resection (ER), surgery and oncological medical treatment 2 .

We report the case of an 80-year-old woman with a subepithelial lesion of the greater gastric curve ( Video 1 ). During endoscopic ultrasound (EUS), it appeared as an oval, hypoechoic homogeneous 29 mm lesion with hypervascularization on contrast-enhanced-EUS, originating from the muscular layer 3 . Fine needle biopsy histology revealed a G1 GIST 4 .

An endoscopic image showing marking of the gastrointestinal stromal tumor by using a linear echoendoscope advanced through the submucosal tunnel with an argon plasma coagulation probe during submucosal tunnel endoscopic resection.Video 1

The GIST was treated by using a submucosal tunnel endoscopic resection (STER) technique with an operative gastroscope (EG-760CT, Fujifilm corp. Tokyo, Japan) and a HybridKnife T-type I-jet (ERBE Elektromedizin, Tuebingen, Germany).

**Fig. 1:**
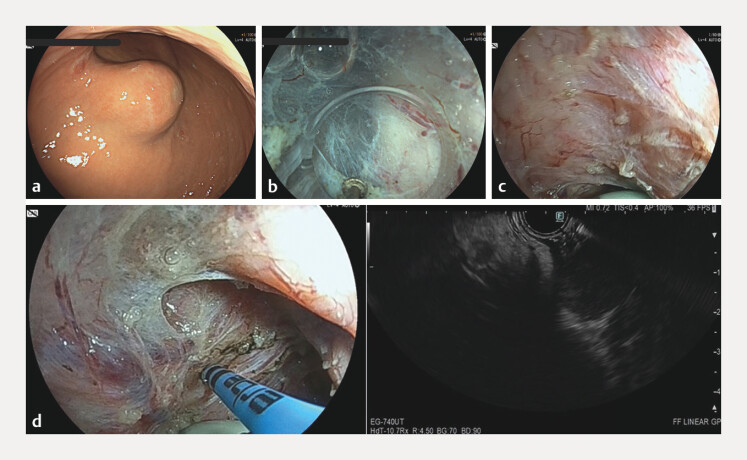
(Panels **a–d** ): Panel **a** : A subepithelial lesion of the lesser curve of the stomach, previously diagnosed as a low-grade GIST, was planned for endoscopic removal with submucosal tunnelling endoscopic resection (STER). Panel **b** : During the creation of the submucosal tunnel, it was difficult to identify the lesion. Panel **c** : To overcome this unexpected issue, an echoendoscope was advanced through the submucosal tunnel to allow the lesion’s identification. Panel **d** : Combined endoscopic and endoscopic ultrasound images of the marking of the lesion’s margins with an argon plasma coagulation probe.

Initially, the lesion was visible as subepithelial bulging. After submucosal tunneling, the lesion was not clearly detectable both from inside and outside the tunnel, as it was located within the muscular layer and covered by muscular fibers. Thus, to identify the GIST, we performed an EUS by advancing a linear echoendoscope (EG-740UT, Fujifilm corp. Tokyo, Japan) through the submucosal tunnel. The GIST was then identified, marked with argon plasma coagulation 30W on the muscular layer ( Fig. 1 , panels **a–d** ) and removed after the selective dissection of the muscular fibers covering the lesion. At the end of the procedure, the tunnel was intact, and the access was completely closed with through-the-scope clips. Histology confirmed the G1 GIST.

To our knowledge, this is the first report of a GIST of the muscular gastric layer treated with STER, requiring intraprocedural identification with EUS performed from within the submucosal tunnel. Lesions originating from the deep gastric wall layer may be at a higher risk of ER failure, also due to possible difficulties in their identification during the procedure 5 . This approach could minimize this risk and ease the procedure, avoiding the need for more invasive treatments such as laparoscopy and endoscopic cooperative surgery.

Endoscopy_UCTN_Code_CCL_1AB_2AC_3AB
